# The Structural Evolution of Semipolar (11−22) Plane AlN Tem-Plate on *m*-Plane Sapphire Prepared by Sputtering and High Temperature Annealing

**DOI:** 10.3390/ma15082945

**Published:** 2022-04-18

**Authors:** Fabi Zhang, Jin Zhang, Lijie Huang, Shangfeng Liu, Wei Luo, Junjie Kang, Zhiwen Liang, Jiakang Cao, Chenhui Zhang, Qi Wang, Ye Yuan

**Affiliations:** 1 Guangxi Key Laboratory of Precision Navigation Technology and Application, Guilin University of Electronic Technology, Guilin 541004, China; zhangfabi@outlook.com (F.Z.); 18811137268@163.com (J.Z.); 18673751825@163.com (L.H.); 2 Songshan Lake Materials Laboratory, Dongguan 523808, China; sfliu@pku.edu.cn (S.L.); kangjunjie@sslab.org.cn (J.K.); cao.jiakang@163.com (J.C.); 3 State Key Laboratory of Artificial Microstructure and Mesoscopic Physics School of Physics, Peking University, Beijing 100871, China; 4 Institute of Physics, Chinese Academy of Sciences, Beijing 100190, China; 5 Dongguan Institute of Optoelectronics, Peking University, Dongguan 523808, China; liangzw@pku-ioe.cn (Z.L.); wangq@pku-ioe.cn (Q.W.); 6 Physical Science and Engineering Division (PSE), King Abdullah University of Science and Technology (KAUST), Thuwal 23955-6900, Saudi Arabia; chenhui.zhang@kaust.edu.sa

**Keywords:** aluminum nitride, high temperature annealing, semi-polarized template

## Abstract

In this work, the epitaxial semipolar (11–22) AlN was prepared on nonpolar *m*-sapphire substrate by combining sputtering and high-temperature annealing. According to our systematic measurements and analysis from XRD, Raman spectra, and AFM, the evolution of crystalline structure and morphology was investigated upon increasing AlN thickness and annealing duration. The annealing operation intensively resets the lattice and improves the crystalline quality. By varying the film thickness, the contribution from the AlN-sapphire interface on crystalline quality and lattice parameters during the annealing process was investigated, and its contribution was found to be not so obvious when the thickness increased from 300 nm to 1000 nm. When the annealing was performed under durations from 1 to 5 h, the crystalline quality was found unchanged; meanwhile, the evolution of morphology was pronounced, and it means the crystalline reorganization happens prior to morphology reset. Finally, the annealing treatment enabled a zig-zag morphology on the AlN template along the sapphire [0001] direction in the plane, which potentially affects the subsequent device epitaxy process. Therefore, our results act as important experience for the semipolar nitride semiconductor laser device preparation, particularly for the epitaxy of microcavity structure through providing the crystalline evolution.

## 1. Introduction

The spread of COVID-19 has intensively boosted the development of high-efficiency sterilization methods; particularly, the pandemic explosion draws considerable attentions into AlGaN-based ultraviolet-C (UVC, <280 nm) luminescent devices due to their efficiency towards coronaviruses [1,2]. Although the AlGaN based UVC-luminescent devices show encouraging superiority in the aspects of fast sterilization, nontoxicity, flexible installability, and portability [3], the wall-plug efficiency (WPE) is still less than 10%, which is still far away from the value of GaN-based blue-LED [3], and it is ascribed to both material and physical reasons. From the view of material, the demand of UVC-transparent meanwhile lattice-match acts as the prerequisite for UVC-LED epitaxy, and as a result, only bulk AlN substrate and AlN/Sapphire template are qualified. However, the directly grown AlN on flat sapphire usually exhibits thread dislocation density (TDD) above 10^10^/cm^2^, and those dislocations would penetrate into the quantum well region and annihilate the emission. Fortunately, such an obstacle was subsequently solved by the epitaxial lateral overgrowth (ELOG) and high-temperature annealing (HTA) techniques, which both effectively suppress the TDD down to the 10^7~8^/cm^2^ level [4,5]. Whereas, from the physical aspect, one of the dominant obstacles is the strong polarization of the nitride semiconductor along the [0001] axis, and it causes a strong quantum-confined stark effect (QCSE), which prohibits the overlap of hole and electron wavefunction [6,7,8,9]. Actually, the QCSE-caused emission degradation has been solved by utilizing nonpolar or semipolar templates; however, such strategies have been limitedly carried out in AlGaN-based UVC-LEDs which suffer much more from the QCSE than in conventional GaN-based blue-LEDs due to increased Al content-enhanced polarization. In addition, in order to ensure the quality of the epitaxial UVC-luminescent device structure, e.g., the microcavity and Bragg reflector region in the laser device, an outstanding crystalline quality is necessary. 

Therefore, exploring the avenue which spontaneously solves above-mentioned issues is of significance for further improving performance, in particular the emission power of AlGaN-based UVC-LEDs. The preparation of high crystalline quality nonpolar or semipolar AlN templates acts as a promising candidate to settle the bottleneck, which has been pursued by several groups [10,11,12,13,14]. Particularly, in order to achieve outstanding crystallinity, the strategy of HTA is preferred rather than ELOG due to the challenge of manipulating epitaxy orientation during the MOCVD growth [13,15,16,17]. However, the systematic investigation on the evolution of strain inside the HTA AlN layer on *m*-plane sapphire is still limited. 

In the present work, by fully utilizing the magneto-sputtering combined with the high-temperature annealing technique, we obtained the single-crystalline semipolar (11−22) AlN template on nonpolar *m*-plane sapphire substrate. Moreover, the structural information was systematically studied to explore the effect from HTA, including the crystallinity as well as morphology. In particular, by varying the thickness and annealing time, the changing of crystallinity and structure were both tracked to conclude the influence from the interface. As a consequence, our results act as the solid and meaningful experience for lateral studies on semipolar UVC-luminescent devices, particularly with specific structures, e.g., microcavity in the laser device. 

## 2. Experiment

The (11–22) plane semipolar AlN template was grown on *m*-plane sapphires by magneto-sputtering, and the high pure aluminum (purity~99.999%) was the target. The sputtering ambient was set as the argon and nitrogen mixture at a ratio of Ar:N_2_ = 1:4, and the flux of Ar and N_2_ were 20 and 80 sccm, respectively. The sputtered thickness was manipulated according to the growth speed under the sputtering power of 3000 W. Moreover, in order to calibrate the thickness, the ellipsometry was also employed. The temperature and pressure during the sputtering process were 700 °C and 9 × 10^−1^ Pa, respectively. The AlN templates were annealed by using a tube furnace at 1700 °C for over 5 h, and the annealing ambient was pure nitrogen (99.99%). The X-ray diffraction-rocking curves of AlN (11–22) and (11-20) planes, 2theta-omega, as well as Phi scan measurements were performed by X-ray diffraction (XRD, Brucker D8 Discovery). The full width at half maximums (FWHMs) were obtained by fitting the curve with Gaussian fitting. Atomic force microscopy (AFM, Veeco Dimension TM 3100) with a typing mode was used to explore the surface morphology of all AlN samples. Raman was performed by using a LabRam HR Evolution microscopic confocal Raman spectrometer from Horiba. Micro-Raman spectroscopy was excited by an unpolarized 532 nm laser.

## 3. Result and Discussion

**Figure 1:**
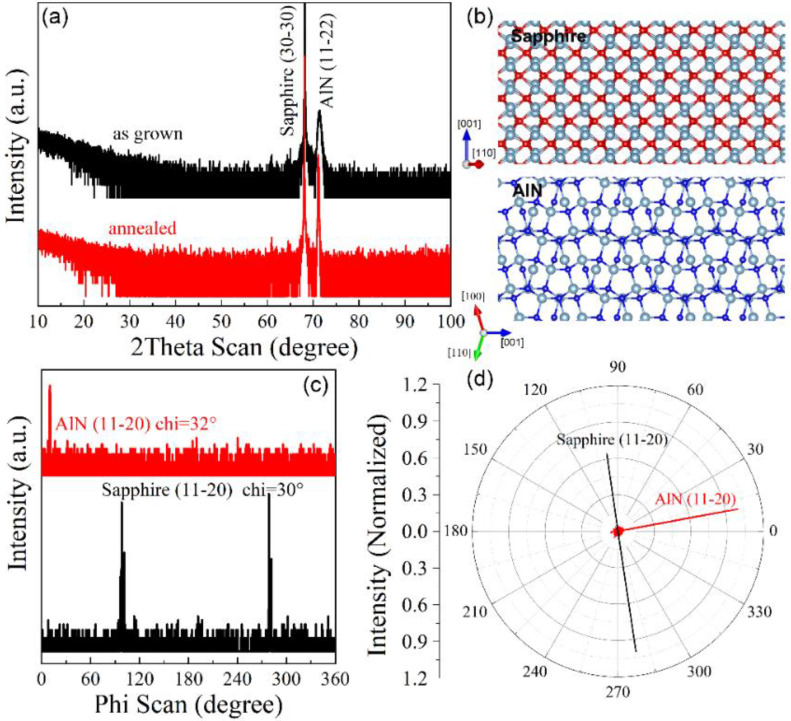
(**a**) XRD 2Theta-Omega scans of as-grown and annealed AlN samples on *m* plane sapphire along the out-of-plane direction; (**b**) The plan-view of crystal-lattice along the sapphire $\left[10\bar{1}0\right]$ and AlN $\left[11\bar{2}2\right]$ directions; XRD (**c**) Phi-dependent and (**d**) polar scans of sapphire (11-20) and AlN (11-20) planes when the chi angle is 32° and 30°, respectively. The thickness and annealing duration of the measured samples are 500 nm and 5 h, respectively.

Figure 1 shows the X-ray diffraction results of as-grown and annealed AlN samples on *m*-plane sapphire in an attempt to explore the epitaxial relation between upper AlN film and *m*-plane sapphire substrate. As shown in Figure 1a, the 2theta-omega scan along the out-of-plane direction indicated that only the sapphire (30-30) and AlN (11–22) planes appeared. Such a phenomenon presents the sign of epitaxial relation between sapphire (30-30) and AlN (11–22) planes. When we explored the structural configuration in the plane, the XRD phi scans were utilized when the Chi were set as 32° and 30° in order to track the (11-20) planes of both AlN and sapphire template. The phi-dependent scanning results are presented in Figure 1c, where one and two diffraction peaks from AlN and sapphire substrate, respectively, were observed when the samples were rotated 360° around the film normal. For a convenient reading, the data were plotted as a polar figure shown in Figure 1d. It is clearly observed that the in-plane component of the AlN $\left[11\bar{2}0\right]$ direction was vertical to the in-plane component of the sapphire $\left[11\bar{2}0\right]$ direction. Such an epitaxial relation is highly in agreement with previous reports [18,19], and it is well maintained even in the high-temperature-treated AlN. Accordingly, the lattice scheme of m-sapphire and (11–22) plane AlN from the out-of-plane direction view is shown in Figure 1b. Moreover, the in-plane component of [0001] directions of both materials were placed vertical to each other, which represents the real case of epitaxial AlN film. 

**Figure 2:**
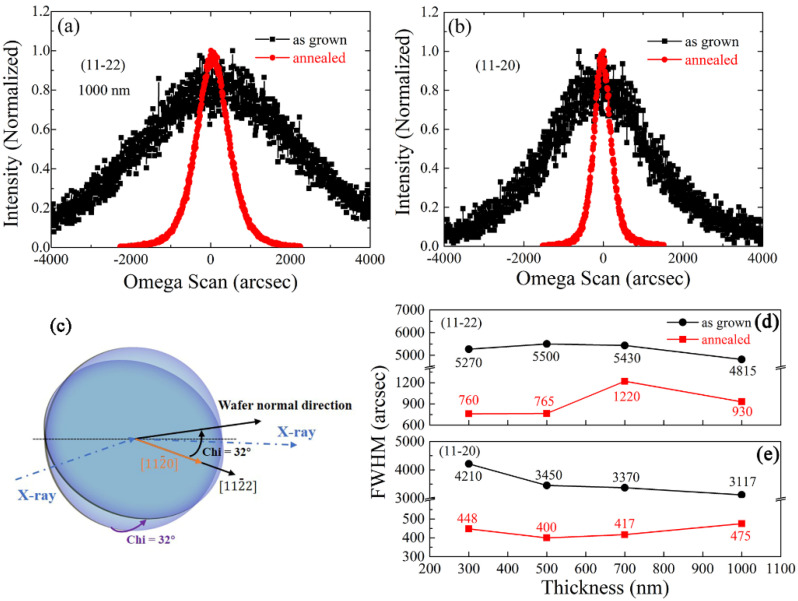
XRD rocking curves of (**a**) (11–22) and (**b**) (11-20) planes of 1000-nm-thick as-grown and annealed AlN samples; (**c**) the geometry of XRD-RC measurements of (11–22) and (11-20) planes; thickness-dependent FWHMs of (**d**) (11–22) and (**e**) (11-20) RC curves. The annealing duration is 5 h.

More detailed results about crystalline quality of as grown and annealed samples are shown in Figure 2. The full width at half maximum (FWHM) of the XRD rocking curve (RC) is always used as a standard criterion to qualify the crystalline quality of single crystal materials. Generally speaking, for conventional *c*-AlN on *c*-Sapphire substrate, (0002) and (10-12) planes were all measured due to the consideration of both out-of-plane and in-plane direction components [20]. Herein, by taking into account the above-mentioned two components, the (11–22) and (11-20) planes were selected for the RC measurements. As presented in Figure 2a,b, the high-temperature annealing operation largely sharpened the RC curves of the 1000-nm thick sample, which means the crystalline lattice was largely reordered. The FWHMs were calculated in order to quantitatively evaluate the annealing effect. Figure 2d,e shows the thickness-dependent FWHMs of (11–22) and (11-20) planes for as-grown and annealed AlN samples, and it was seen that the as grown samples with different thicknesses all exhibited FWHMs above 3000 arcsec along both directions. The high-temperature treatment effectively drove the blend of crystalline boundary and dislocation annihilation, causing the recrystallization of as-sputtered samples, and the FWHMs obviously decreased. Particularly, it is worth noting that after annealing the FWHM values of (11-20) were smaller than the ones of (11–22) in all samples, which means that the lattice order along the in-plane $\left[11\bar{2}0\right]$ direction was better than that along the out-of-plane $\left[11\bar{2}2\right]$ direction. Upon increasing thickness, two series of FWHMs did not present obvious thickness dependent feature, which indicates that the AlN-sapphire interface did not contribute dominantly to recrystallization. When compared with as-grown samples, the FWHMs of annealed samples did not present obvious thickness-dependent features. 

**Figure 3:**
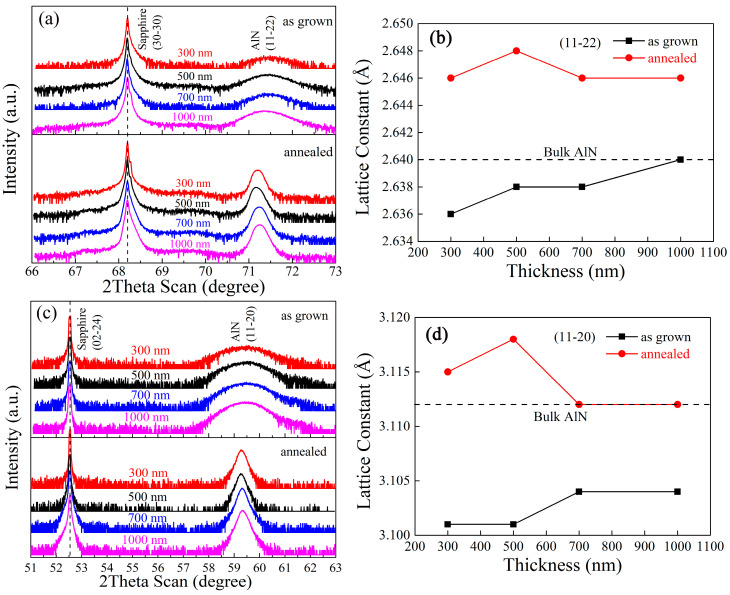
(**a**,**c**) The 2theta-Omega scans along different crystalline directions of as-grown and 5 h-annealed samples with various thicknesses; the lattice constants of (**b**) (11–22) and (**d**) (11-20) planes calculated from the 2Theta-Omega curves as a dependence of thickness.

Figure 3 shows the 2theta-omega scans along the $\left[11\bar{2}2\right]$ and $\left[11\bar{2}0\right]$ directions, and the results act as the direct evidence of lattice evolution. As shown in Figure 3a,c, the diffraction peaks of as-grown samples were like drum rather than peak, which confirms the existence of plenty of disorder in the lattice. Calibrated by the sapphire diffraction peak, the diffraction peaks of AlN with various thicknesses were placed together for a comparison. The lattice parameters calculated from the 2theta-omega results are shown in Figure 3b,d according to the Bragg equation:

(1) 2dsinθ=nλ

where the *λ* = 1.54059 Å is the wavelength of the X-ray during the measurement. For a comparison, the ideal lattice parameters of bulk AlN are marked as dashed lines in the figure as a reference. Before annealing, the lattice constants of both planes were smaller than the ideal values; moreover, it rose and gradually approached the strain-relax case upon increasing thickness. Such a result confirmed the role of an interface that introduces the strain or lattice distortion in as-grown case. Interestingly, the annealing treatment successfully drove the lattice parameters to be larger than the ones in the bulk case, indicating that the annealing also resets the strain statue in addition to the crystalline order. The lattice distortion indicated a strain evolution from tensile strain to compressive strain caused by the high-temperature annealing operation. 

**Figure 4:**
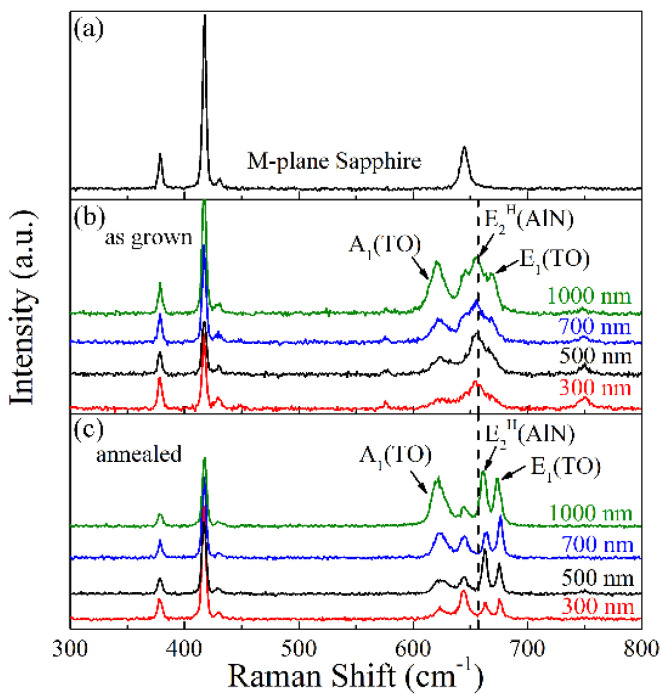
The Raman spectra of (**a**) referenced *m*-sapphire substrate, (**b**) as-grown, and (**c**) 5 h-annealed AlN samples with different thicknesses.

In addition to XRD results, the Raman spectroscopy is also a powerful tool to detect the crystalline information in the lattice. As a reference, a Raman spectrum of *m*-plane sapphire is present as shown in Figure 4a. According to previous studies, we can see the phonon scatterings from the AlN ranged from 600 cm^−1^ to 700 cm^−1^. After subtracting the E(g) Raman signal from *m*-sapphire at 645 cm^−1^, for the 300 nm as-grown AlN film, the phonon-scattering peak of E_2_^H^ was the most intensive, while only the signs of A_1_(TO) and E_1_(TO) modes were present. The high-temperature annealing fully activated the A_1_(TO) and E_1_(TO) signals due to the improvement of crystalline quality [21]. In addition, when compared with the as-grown sample, the E_2_^H^ peak exhibited a blueshift from 654 cm^−1^ to 663 cm^−1^ in the annealed sample. Such a Raman blueshift again confirmed a transition from tensile strain in the as-grown sample into compressive strain in the annealed sample [15], which is consistent with the XRD description in Figure 3. With increasing AlN thickness, the A_1_(TO) peak became more and more intensive and separated in the as-grown sample. Despite the intensity of the E_1_(TO) mode being continuously enhanced as the thickness rose, the signal still mostly merged with the E_2_^H^ signal. The annealing successfully separated the three phonon-scattering peaks when the thickness gradually increased. Actually, in addition to the E_2_^H^ mode in the 300-nm sample, the other two AlN-scattering modes both showed blueshift behavior resulted from the annealing, again verifying the evolution of strain statue.

**Figure 5:**
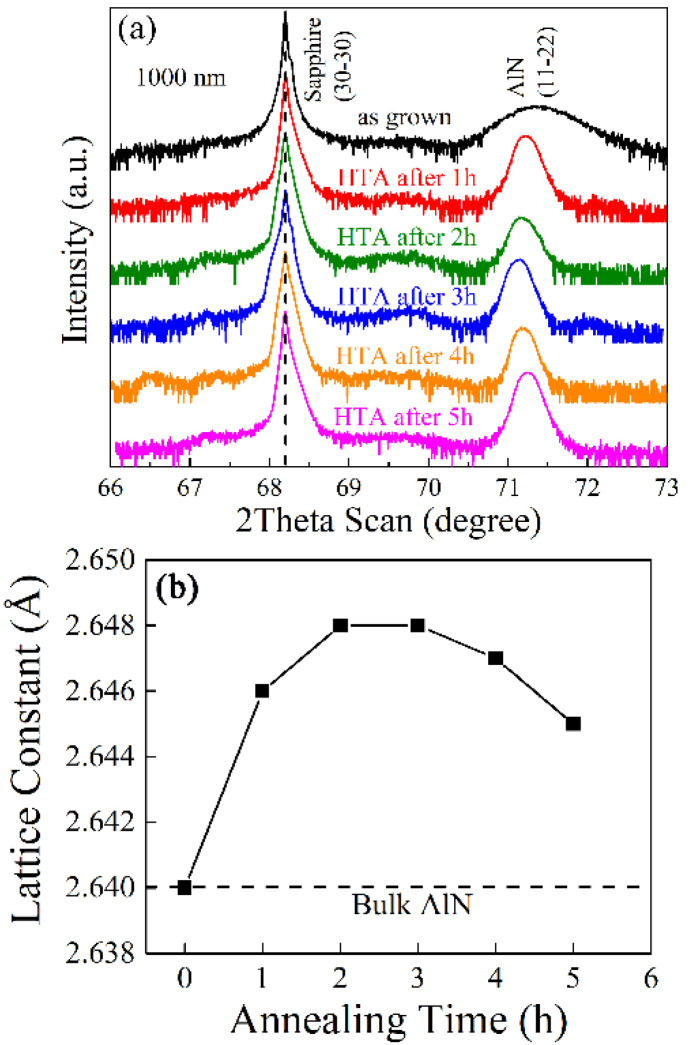
(**a**) 2Theta-Omega scans and (**b**) corresponding calculated lattice parameters of out-of-plane (11–22) plane when the 1000 nm-thick samples are annealed under different durations.

The high-temperature operation largely reordered the lattice matrix during the annealing process. Therefore, the evolution of lattice constant could be captured during the annealing process. In order to track the crystallinity improvement, the 2theta-omega scans of samples annealed from 1 to 5 h are shown in Figure 5. It is clearly seen that the annealing time as short as only one hour was more than enough to sharpen the diffraction peak of (11–22) plane, and it suggests that the crystalline quality reset happens very soon when the annealing temperature is above the threshold of lattice reorder. However, as the annealing goes on, the position of the (11–22) diffraction peak successively experienced a shift during the 5 h, and it indicated that the lattice expands in the initial 2 h and subsequently continuously shrinks to 2.645 Å until 5 h, which is observed in Figure 5. Anyway, the lattice parameter is larger than the value of bulk AlN.

**Figure 6:**
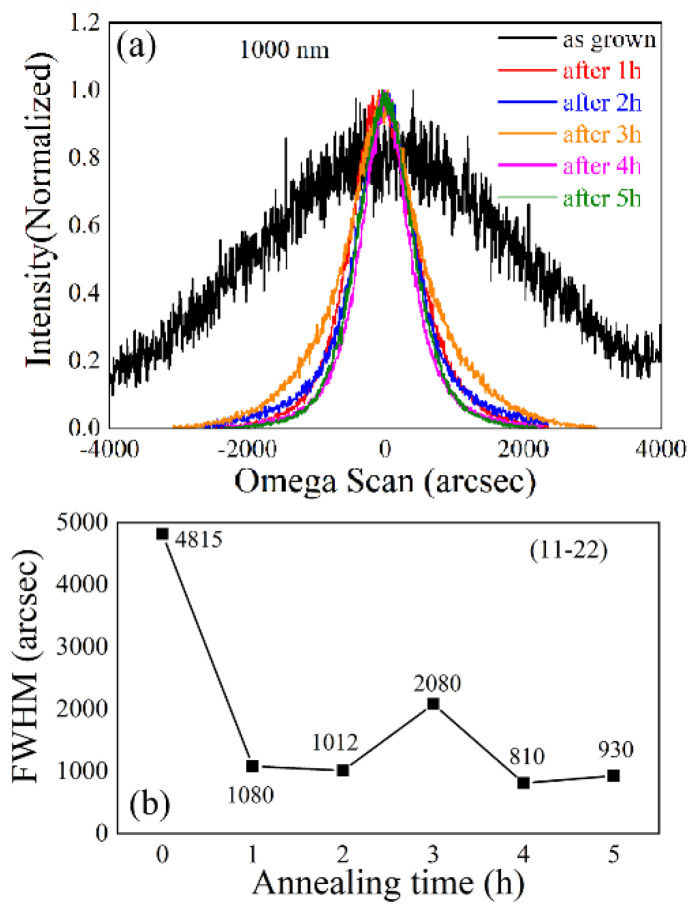
(**a**) XRD rocking curves and (**b**) corresponding FWHMs of (11–22) plane of 1000 nm-thick AlN annealed under different durations.

**Figure 7:**
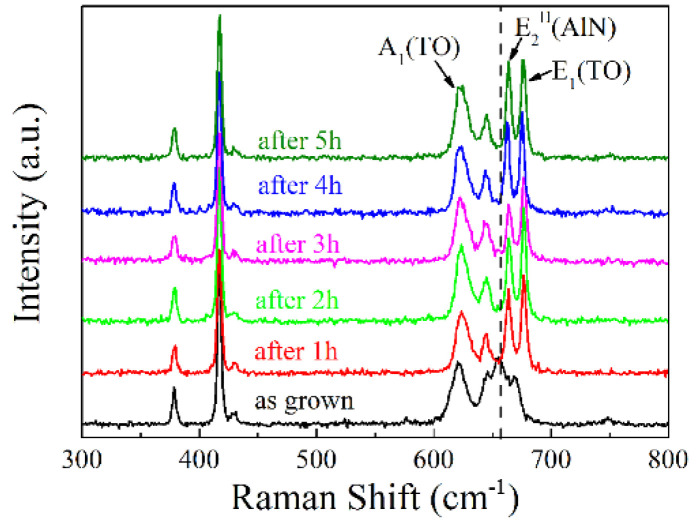
The Raman spectra of 1000 nm-thick AlN samples annealed under different durations.

The crystalline quality evolution upon increasing annealing duration was reexplored by XRD RCs as shown in Figure 6. As shown in Figure 6, the crystalline quality evaluated by RC curves did not gradually improve in the time scale of hours, and the 1 h annealed sample showed the (11–22) plane RC FWHM as low as 1080 arcsec. However, when the annealing duration was 3 h, the value increased back up to 2080 arcsec. When a larger annealing duration was performed above 3 h, the FWHM returned down to 810 and 930 arcsec when it was annealed under 4 and 5 h, respectively. In addition to the RC results, the Raman spectra were also measured to explore the stress evolution as plotted in Figure 7. Actually, corresponding to the RC results, only 1 h annealing was more than enough to expose the incisive E_2_^H^ and E_1_(TO) signals, indicating that only one-hour-high temperature treatment successfully recrystallized the sample.

**Figure 8:**
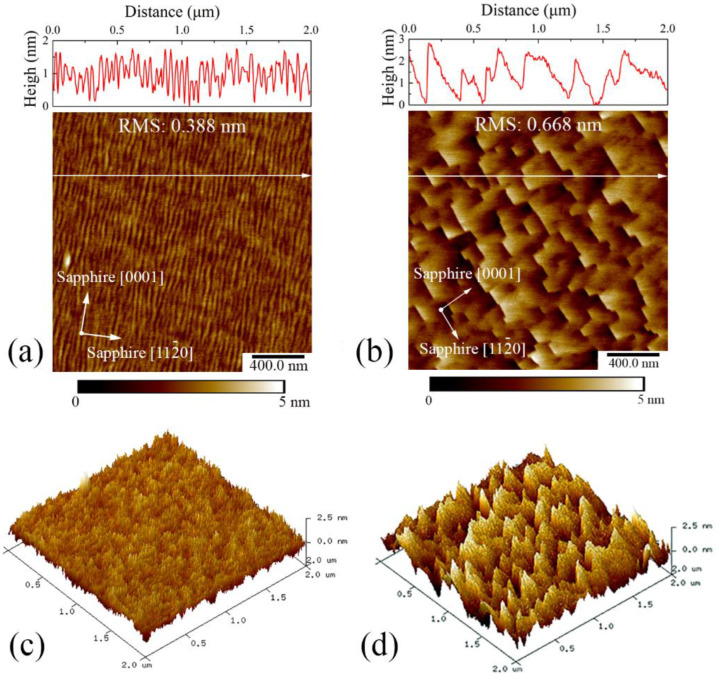
Top and 3D-viewed AFM images of (**a**,**c**) as grown and (**b**,**d**) 5-h annealed AlN samples. The AlN thickness is 500 nm.

In order to explore the surface morphology of semipolar AlN before and after 5 h high-temperature annealing, the 2 × 2 μm^2^ AFM measurements were carried out as shown in Figure 8. Before the annealing, the as-grown sample presented stripe-like morphology, which is vertical to the sapphire *c* axis direction, due to the orientation of AlN crystallization. According to previous studies, such an orientation-preferential growth mode has been observed by the TEM method [15,16], and it originates from the anisotropic lateral growth rate of the grains [22]. Moreover, such a striation morphology which is along the sapphire [0001] direction seems to be strongly related with the grown temperature [15], and our preparation condition just agreed with the qualification. The width and vertical distance of one wire-like structure are ~20 nm and ~1.4 nm, respectively. The calculated root mean square (RMS) of the as-grown sample is 0.388 nm. However, after the annealing, the morphology became square-like, which originates from the surficial atom reorganization. The top view top-viewed orthogonal edge perfectly represents the [0001] direction of the substrate [23]. Despite that the periodical square-piece replaced the original striation shape, the RMS slightly increased to 0.668 nm, and the height of the step was in the range of 1 to 3 nm. When the morphology was cross-sectionally cut along the sapphire [0001] direction, the angle between exposed surface and the horizontal plane was 50°, indicating that the exposed surface plane was most probably (11-2-1), which is principally 49.34° with the (11–22) plane.

**Figure 9:**
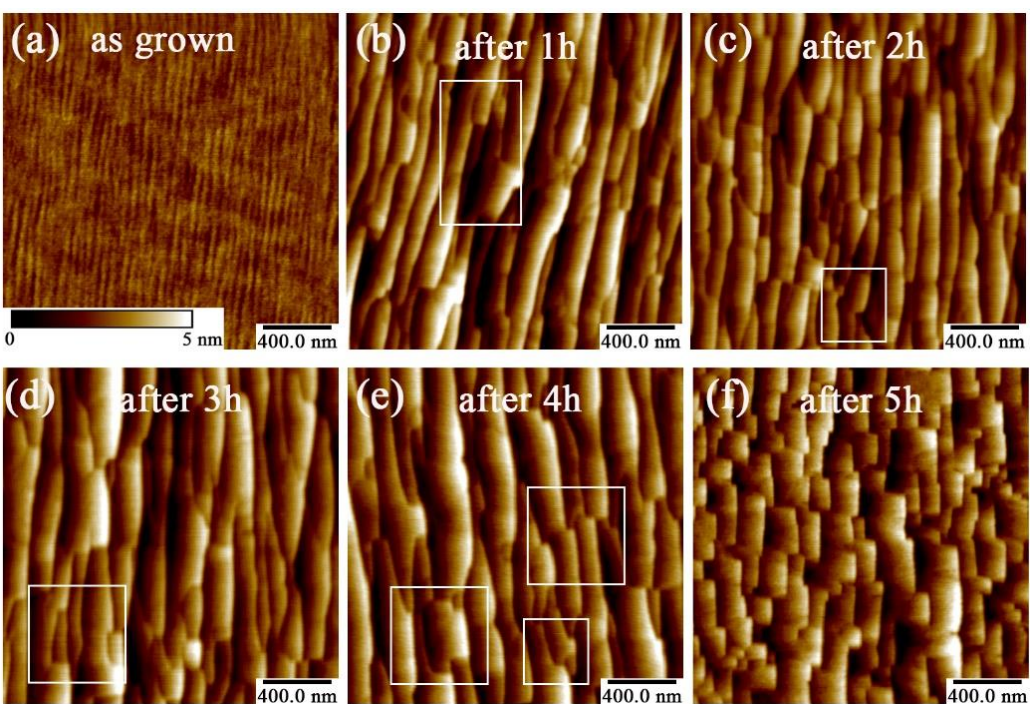
The AFM images of (**a**) as-grown and annealed AlN templates under (**b**) 1 h, (**c**) 2 h, (**d**) 3 h, (**e**) 4 h, and (**f**) 5 h. The AlN thickness is 500 nm.

Figure 9 presents the morphology evolution upon increasing annealing duration by AFM. As the figure shows, in the beginning one hour, the morphology was largely reset. The thin ripples started to aggregate and form the step morphology, which was somehow step-bunching-like. The step was parallel with the $\left[11\bar{2}0\right]$ direction of sapphire substrate, and the step height was 3.3 nm. Likewise, square-like edge started to appear somewhere as shown by the white mark, which reprints the lattice structure of the AlN film along the in-plane direction. Upon increasing the annealing duration up to 3 h as shown in Figure 9d, the morphology did not change. Even the step height was maintained as 2.65 and 3.72 nm for 2-h and 3-h annealing samples. However, when the annealing duration was up to 4 h, the zig-zag-like edge area spread with the step-height of 4.06 nm, as shown in Figure 9e. Until 5 h, the surface morphology totally became mosaic-like. It is worth noting that as the annealing duration increased, the RMSs were 0.316 nm, 1.02 nm, 0.776 nm, 0.958 nm, 0.949 nm, and 0.916 nm in the as-grown sample, 1-h, 2-h, 3-h, 4-h, and 5-h annealed samples, respectively. Thus, despite that the RMS was obviously reset by the annealing operation, the annealing duration did not seem contribute to the RMS value.

Actually, if only the morphology itself was focused on, it was really not smooth enough, and seems even impossible to be used in epitaxy. However, despite the morphology being step-like, the vertical distance of each step was ~3 nm, which is comparable with the step of conventional step-bunching morphology whose vertical distance is as large as several nanometers in previous device epitaxy [24]. Therefore, it acts as an ideal platform to perform subsequent epitaxial growth.

## 4. Conclusions

In summary, we successfully obtained the epitaxial semipolar (11–22) AlN on nonpolar *m*-sapphire substrate by combining sputtering and high-temperature annealing. The high-temperature annealing treatment intensively improved the crystalline quality through reordering both lattice and stress. Through varying the AlN thickness, no obvious changing was observed in both crystalline quality and strain state, therefore negating the huge contribution from the sapphire-AlN interface during the annealing process. Upon increasing the annealing duration from 1 to 5 h, although the crystalline reorder process was carried out in the first annealing hour, the morphology gradually changed into square-like zig-zag morphology upon increasing the annealing duration up to 5 h. As a consequence, our results are of importance as basic information for subsequent semipolar device epitaxy.
